# A trial for the use of qigong in the treatment of pre and mild essential hypertension: a study protocol for a randomized controlled trial

**DOI:** 10.1186/1745-6215-12-244

**Published:** 2011-11-21

**Authors:** Ji-Eun Park, Yan Liu, Taeseob Park, Sanghoon Hong, Jung-Eun Kim, Tae-Hun Kim, Ae-Ran Kim, So-Young Jung, Hyoju Park, Sun-Mi Choi

**Affiliations:** 1 Department of Medical Research, Korea Institute of Oriental Medicine, Daejeon, South Korea; 2 University of Science & Technology, Daejeon, South Korea; 3 Qigong Treatment Center, Dongeui Medical Center, Pusan, South Korea; 4 Department of Internal medicine, College of Oriental medicine, Dongeui University, Pusan, South Korea

## Abstract

**Background:**

Hypertension is a risk factor for cardiovascular disease, and the prevalence of hypertension tends to increase with age. Current treatments for hypertension have side effects and poor adherence. Qigong has been studied as an alternative therapy for hypertension; however, the types of qigong used in those studies were diverse, and there have not been many well-designed randomized controlled trials.

Our objectives are the following: 1) To evaluate the effects of qigong on blood pressure, health status and hormone levels for pre- or mild hypertension. 2) To test the methodological appropriateness of this clinical trial and calculate a sample size for future randomized trials.

**Methods:**

Forty subjects with pre- or mild hypertension will be randomized to either the qigong exercise group or the non-treated group. Participants in the qigong group will conduct qigong exercises 5 times per week for 8 weeks, and participants in the non-treated group will maintain their current lifestyle, including diet and exercise. The use of antihypertensive medication is not permitted. The primary endpoint is a change in patient blood pressure. Secondary endpoints are patient health status (as measured by the SF-36 and the MYMOP2 questionnaires) and changes in hormone levels, including norepinephrine, epinephrine, and cortisol.

**Discussion:**

This study will be the first randomized trial to investigate the effectiveness of qigong exercises for the treatment of pre- and mild hypertension. The results of this study will help to establish the optimal approach for the care of adults with pre- or mild hypertension.

**Trial registration:**

Clinical Research Information Service KCT0000140

## Background

Hypertension is a risk factor for future cardiovascular events, including heart attack, heart failure, and stroke, which are the most frequent causes of death in developed countries [1,2]. Hypertension affects approximately one billion individuals worldwide, and the prevalence of hypertension tends to increase with age [3]. In Korea, hypertension affects more than 30% of people over the age of 30 and up to 65% of people over 65 [4].

Current interventions for hypertension include sodium restriction, pharmacological management, and lifestyle modifications, such as stress management and exercise. However, in clinical practice, hypertension can be difficult to control, due to poor adherence to prescribed interventions [5]. Lifestyle interventions are difficult to achieve and maintain, and antihypertensive medications present risks of side effects.

Qigong is a common healing technique in Oriental medicine and has been used clinically for preventing and curing disease, as well as for improving and maintaining overall health [6]. It has been claimed that qigong can foster health and healing by promoting the smooth flow of qi throughout the body so that the body can heal itself [7]. Qigong includes two concepts: qi, the vital energy of the body, and gong, the training or cultivation of qi. Medical qigong is divided into internal and external components. Internal qigong consists of exercises, including breathing, meditation, focus of intention and rhythmical movements, while external qigong is performed by a trained practitioner in order to deliver qi energy to the patient [8]. It is generally accepted that internal qigong is the more beneficial aspect for the promotion of good health [9].

Qigong has been found to have effects on various conditions, such as cancer [10-12], pain [13] and diabetes [14]. Qigong has also been studied as an alternative therapy for controlling blood pressure. Previous such research has concluded that patients with essential hypertension practicing qigong for less than 1 year had improved blood pressure (BP) control compared with non-treated controls [7].

However, well-designed rigorous randomized controlled trials (RCTs) related to the effects of qigong on BP control are still scarce [15]. Although most qigong trials have targeted essential hypertension, the stages of hypertension exhibited by study subjects were diverse, and some studies did not describe each patient's exact blood pressure. In addition, there has been no trial assessing the effects of qigong on pre-hypertension. To assess the effects of qigong for pre- and mild hypertension through a high-quality RCT, a pilot study to evaluate qigong should be conducted.

The aims of this pilot study are the following: (1) to evaluate the effects of internal qigong on blood pressure, health status and hormone levels in patients with pre- or mild hypertension and (2) to test the methods and calculate a sample size for future randomized trials to investigate further the clinical value of qigong in controlling hypertension.

## Methods/Design

### Study design

**Figure 1:**
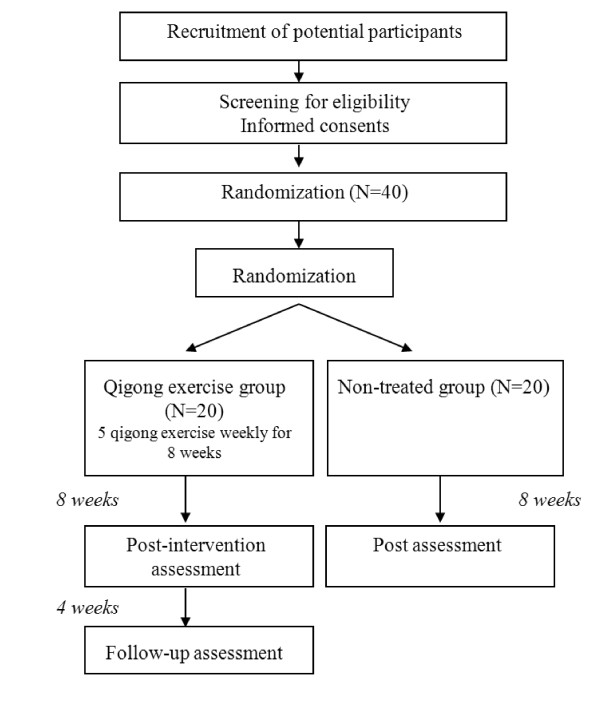
**Flowchart of the study design**.

The study is a 2-parallel group, randomized controlled trial to compare qigong with no treatment for pre- and mild hypertension on the following outcomes: blood pressure, Health Related Quality of Life (HRQOL), clinical symptoms and hormone levels. The study design is depicted in Figure 1.

### Ethics

The protocol has been approved by the institutional review boards of Dongeui University, where the study will take place. This study has been registered with the 'Clinical Research Information Service', Republic of Korea, which is a registry in the WHO Registry Network [16].

This study will be monitored by an independent data and safety monitoring board. Advertisements for voluntary subjects will be printed in local newspapers and posted on notice boards in the local community. All of the participants will provide written informed consent.

### Participants

#### Inclusion criteria

The inclusion criteria are as follows: (1) patient age between 19 and 65 years, (2) systolic blood pressure between 120 and 159 mm Hg and/or diastolic blood pressure (DBP) between 80 and 99 mm Hg as measured in a sitting position, and (3) the absence of objective signs of hypertension end-stage disease.

#### Exclusion criteria

The exclusion criteria are as follows: (1) serious symptomatic cardiac disease, including previous myocardial infarction, angina or heart failure; (2) previous transient ischemic attacks or stroke; (3) secondary hypertension; (4) taking medications that could affect hypertension, such as stimulants, central nervous system depressants; (5) necessity of antihypertensive medications; (6) concomitant illness that precludes participation, including diabetes, cancer, infectious disease, renal failure; (7) pregnancy or the possibility of pregnancy; (8) moderate to high risk of cardiac complications during exercise; or (9) inability to comprehend and complete the study assessments or comply with the study instructions (Table 1).

**Table 1:** Eligibility criteria

Inclusion criteria	Exclusion criteria
19-65 years of age	Serious symptomatic cardiac disease, including previous myocardial infarction, angina or heart failure

Pre- or Stage 1 hypertension -Systolic blood pressure between 120 and 159 mm Hg - Diastolic blood pressure between 80 and 99 mm Hg	Previous transient ischemic attacks or stroke

No objective signs of hypertension end-stage disease	Secondary hypertension

	Taking medications that could affect hypertension

	Necessity to continue antihypertensive medications

	Concomitant illness that precludes participation, including diabetes, cancer, infectious disease, renal failure

	Pregnancy or chance of pregnancy

	Moderate to high risk for cardiac complications during exercise

	Inability to comprehend and complete study assessments or comply with study instructions

#### Participant recruitment

We will recruit 40 participants; all participants will provide written consent before participation in the study. The clinical research coordinator (CRC) will initially screen by telephone each potential participant against the inclusion and exclusion criteria. Willing potential participants will be given an appointment at the center for the qigong trial. A screening log will be maintained to record the inclusion/exclusion criteria for all those participants. The CRC will briefly explain the purpose of the study and ask if they have an interest in participating. The CRC will also record basic demographic and medical information.

#### Sample size

There are no previous clinical trials to evaluate the effect of qigong on pre- or mild hypertension. Therefore, this study is designed as a pilot study to calculate the appropriate sample size for future randomized clinical trials. Each group will include 20 participants, which is the minimum sample size for evaluating the effect of qigong [17,18].

#### Randomization and allocation concealment

The subjects admitted to the trial will be randomized to either the qigong group or the non-treated group. Participants will be randomized before the first treatment by an online centralized randomization service. Randomization will be stratified by age and sex.

Allocation concealment will be ensured, as the service will not release the randomization code until the patients are recruited into the trial after all baseline measurements are completed.

#### Collection of demographic and lifestyle information

Each patient's medical history, including current medication, surgical history, and the presence of other diseases, will be recorded at baseline. Lifestyle factors, including diet, exercise, smoking, and drinking alcohol, will also be documented. To investigate other risk factors for hypertension, height, weight, waist and hip circumference, and other demographic information will also be measured. Body-fat percentage will be estimated by measuring bioelectrical impedance. A questionnaire for syndrome differentiation will be used based on general condition, sweating, pain, sleeping, breathing, skin and pulse.

#### Blinding

Outcome assessors will be blind to group allocation and will not be involved in providing intervention. However, it will be difficult to blind the participants because the control group will not receive any intervention.

### Intervention

#### Qigong exercise

Although there have been several qigong trials for hypertension, the type of qigong used in those trials was diverse, and there has been no standardized qigong exercises for the treatment of hypertension. We have developed a qigong protocol for the treatment of hypertension based on discussions with three qigong professionals.

Qigong will be taught by an instructor with expertise in qigong. The participants will visit the clinic and take 3 qigong classes per week for 8 weeks. Each qigong class lasts 30 minutes, including a warm-up (5 min) and a cool-down (5 min) portion. In addition, the subjects will be asked to practice qigong at home for 30 min more than 2 times a week for the duration of the study. The purpose of qigong exercise is to improve kidney and liver function, to improve blood circulation and to lower blood pressure through calming (Table 2).

**Table 2:** Composition of Qigong

Stage	Stage	Motions
Warm-up	1	Meditate for 1 minute, swing your arms and shoulders and flex your waist, knees and ankles.
1^st^	1	Lift both hands overhead, locking your fingers together while bending and extending your knees.
	2	Lean your body to the right while breathing in, and stand straight, while breathing out. Repeat 4 times to the left and right, alternating.
2^nd^	1	Advance your left foot, and lower your right hand from the left of your head to your right leg drawing an oblique line quickly. Repeat this 8 times to the left and right, alternating.
3^rd^	1	Advance your right foot, lift your right arm and support your right elbow with the back of your left hand.
	2	Lower your body quickly while bending your knees and clenching your right fist. Repeat this 8 times to the left and right, alternating.
4^th^	1	Advance your left foot and put your left hand on your left knee. Put your right hand on your left hand and push down as much as possible. Lift up right hand to the right. Repeat this 8 times to the left and right, alternating.
5^th^	1	Put your feet apart and relax your wrists and lift your hands slowly forward.
	2	Lower your hands while bending your knees. Repeat this 10 times.
6^th^	1	Support your back with your hands and lean back.
	2	Bend your waist forward and sweep the back of your legs with your hands.
	3	With straight waist, lift your hands overhead while breathing in and lower them, while breathing out. Repeat this 8 times.
7^th^	1	Put your feet apart and twist your body backward to the left.
	2	Reverse your palms and gather them on your forehead.
	3	Look straight ahead slowly again. Repeat this 8 times to the left and right, alternating.
8^th^	1	Lift your arms to the right.
	2	Lower your body while crossing your left leg backward, and stand straight. Repeat this 10 times to the left and right, alternating
9^th^	1	Bend your knees and lift your arms in parallel and make a circle.
	2	Extend your knees and spread your arms. Twist your upper body backward to the right.
	3	Stand straight while breathing out. Repeat this 8 times to the left and right, alternating.
10^th^	1	With your left foot advanced, lift your right arm up. Tap the area of your liver with your left hand 14 times. Repeat this movement 2 times.
Cool-down	1	Bend your knees and lift up your arms shoulder high. Put your feet apart while breathing in, and gather your feet while breathing out. Repeat this movement 10 times to the left and right, alternating.
	2	Concentrate your mind on an acupoint KI1 on the bottom of your foot for 1 minute.

The compliance of the subjects will be assessed in terms of the number of qigong classes attended and the number of home repetitions per week. No antihypertensive medications or medications that affect blood pressure will be permitted during the study.

#### Non-treated group

The control group will receive no instruction in qigong exercise. They will be asked to maintain their routine lifestyle, including diet, exercise, work and other activities.

### Outcome measures

#### Primary

The primary outcome will be changes in patient blood pressure before and after qigong exercises. Blood pressure will be taken at every visit. The subjects will be fitted with a BP cuff on the right arm and allowed to sit quietly for 5 minutes. A trained research nurse will measure the blood pressure carefully with the subject in the seated position 3 times after resting for at least 10 min in a temperature-controlled room. Three measurements will be carried out at 5-min intervals. The mean of these 3 measurements will be used in the data analysis.

Subjects will be instructed to wear loose and comfortable clothing. The investigators will refrain from wearing laboratory coats during all sessions, as the use of such apparel has been associated with increased BP in some patients (white-coat hypertension).

#### Secondary

Subjects will be asked to complete two questionnaires, specifically, the MOS Short Form 36-item (SF-36) (Korean version) and the Measure Yourself Medical Outcome Profile (MYMOP2). These surveys were selected based on the aims of the trial and on their clinical relevance and feasibility. The SF-36 is a widely used, reliable, valid and responsive measure of outcome health survey. The SF-36 consists of eight scales: physical function, role-physical, bodily pain, general health, mental health, role-emotional, social function, and vitality.

The MYMOP is a patient-generated outcome questionnaire, which is problem-specific but includes general well-being. MYMOP aims to measure the outcomes that the patient considers to be most important. MYMOP is applicable to all patients who present with symptoms; these symptoms can be physical, emotional or social [19].

Heart rate will be assessed on every visit, and questionnaires will be administered at weeks 0, 4 and 8. Changes in norepinephrine, cortisol, renin, angiotensin, total cholesterol, and triglycerides will be examined through blood tests at weeks 0 and 8. Blood samples will be taken in the morning after an overnight fast to achieve standard conditions with respect to dietary intake.

#### Follow-up

Four weeks after completion of the 8 weeks of qigong exercise, blood pressure, heart rate, and the SF-36 and MYMOP2 questionnaires will be evaluated. The follow-up assessment is designed to evaluate the long-term effect of qigong exercise.

#### Data analysis

Collected data will be recorded on standardized forms. Descriptive statistics will be calculated for dependent and independent variables. This analysis will include summary statistics of demographic information and outcome measures.

The primary analysis of the data will be undertaken using the principle of intent-to-treat (ITT). Our ITT analysis will include all participants, including those who are not fully compliant and those missing outcome data. We will evaluate the effectiveness of qigong exercise and no treatment on blood pressure at 8 weeks using an analysis of covariance (ANCOVA).

Demographic and clinical characteristics of subjects in the qigong and the non-treated group will be compared upon admission using a 2-sample t-test (continuous data) and chi-square analysis (normal data). Nonparametric methods will be used when assumptions of normality are violated. To control for baseline differences between groups, variables that are significantly different at baseline will serve as covariates in the analyses. Data will be analyzed on an intention-to-treat basis. Software will include the Access and SAS version 9.1 programs. The alpha level will be set at < 0.05.

Adverse events will be reported to the data and safety monitoring board. Adverse events will be recorded when participants present with complaints at any time and will be based on the questioning of participants. Serious adverse events will be recorded as required by the International Conference on Harmonization Guideline E2A. We will submit the results of the trial for publication in an appropriate journal irrespective of outcome. We will report the trial in accordance with the CONSORT statements.

## Discussion

The primary end point for this study is the change in patient blood pressure from baseline to 8 weeks post-therapy. Eight-week changes in hormone levels and health status, as measured by SF-36 and MYMOP2, are secondary end points. The primary comparison is between participants randomly assigned to qigong exercises versus no intervention. Subgroup analyses for age, gender and baseline BP will be conducted by including each classification.

Many qigong researchers consider that the qigong effects on the human body are systemic without targeting one particular element, such as BP [7]. Therefore, we will use the MYMOP 2 and the SF-36 questionnaires, which can measure the quality of life, health status and the severity of subjective symptoms, as outcomes for this study.

The standard mercury sphygmomanometer is the most common device used to measure blood pressure [2]. The accuracy of automatic blood pressure measurement is still controversial [20,21]; therefore, the mercury sphygmomanometer is considered to be the 'gold standard' for measuring blood pressure. To avoid the white-coat effect [22], the CRC will not wear a white coat during this clinical study.

This study will be the first randomized trial to investigate the effectiveness of qigong exercises for the treatment of pre- and mild hypertension. The main focus of this trial is to investigate the role of qigong in reducing the blood pressure of patients with pre- or mild hypertension. This trial will contribute information needed to begin answering important clinical questions regarding the effects of qigong in lowering the blood pressure of patients with pre- and mild hypertension. Although the trial is not sufficiently powered to determine the effectiveness of qigong on hypertension, we will compare the effect size between groups in order to form larger-scale trials in the future.

## Competing interests

The authors declare that they have no competing interests.

## Authors' contributions

JEP conceived of the project, led the design, and wrote the first draft of this manuscript. YL and TSP participated as qigong experts. SHH, JEK, THK, ARK, SYJ, HJP and SMC provided technical advice and wrote the relevant sections of the manuscript. All authors participated in the trial design, commented on drafts of this paper, and read and approved the final manuscript.
